# Aqueous Extracts of *Carica papaya* Embryogenic Callus Kill *Entamoeba histolytica* Trophozoites and Orally Protect against the Development of Amoebic Liver Abscesses in Hamsters

**DOI:** 10.1007/s11686-025-01071-6

**Published:** 2025-06-11

**Authors:** Cynthia Guzmán, María Luisa Villareal-Ortega, Nelly Villalobos, Anabel Ortiz-Caltempa, Marisela Hernández, Mario Néquiz-Avendaño, Luisa-Carolina González-Ramírez, Gladis Fragoso, Edda Sciutto, César Díaz-Godínez, Julio César Carrero

**Affiliations:** 1 Biotechnology Research Center, Universidad Autónoma del Estado de Morelos, Cuernavaca, Morelos Mexico; 2 Department of Immunology, Instituto de Investigaciones Biomédicas, Universidad Nacional Autónoma de México, Circuito Mario de La Cueva s/n, C.U, Coyoacán, Mexico City, 04510 Mexico; 3 Department of Pathology, Faculty of Veterinary Medicine and Zootechnics, Universidad Nacional Autónoma de México, Mexico City, Mexico; 4 Laboratory of Immunopathology, Department of Experimental Medicine, Facultad de Medicina, Hospital General de México, Universidad Nacional Autónoma de México, Mexico City, Mexico; 5 Investigation Group “Análisis de Muestras Biológicas y Forenses”, Clinical Laboratory Career, Faculty of Health Sciences, Universidad Nacional de Chimborazo, Riobamba, Ecuador

**Keywords:** *Entamoeba histolytica*, Amoebic liver abscess, *Carica papaya*, Aqueous extracts, Anti-amoebic, Treatment

## Abstract

**Purpose:**

This study evaluated the anti-amoebic properties of aqueous extracts of two *Carica papaya* callus clones, Wild Type (Pcc-WT-AE) and KETc7-expressing (Pcc-KETc7-AE) clones, in in vitro and in vivo assays.

**Methods:**

*E. histolytica* trophozoites cultures were exposed for 24 h to varying concentrations of the *C. papaya* aqueous extracts, and their viability and IC_50_ determined by MTT assays. In in vivo studies, golden hamsters were infected intraportally with *E. histolytica* trophozoites and orally treated with the *C. papaya* aqueous extracts for 7 days. The animals were sacrificed on day 8, and the development of ALA was recorded. Comparisons were made against metronidazole (MTZ).

**Results:**

Both extracts statistically reduced trophozoite viability at 24 h in a dose-dependent manner in vitro. Pcc-KETc7-AE showed activity to the same extent as MTZ (IC_50_ 36.08 µg/ml vs. 33.54 µg/ml, respectively), whereas Pcc-WT-AE exhibited less efficient but significant activity (IC_50_ 113.4 µg/ml). Cell death analysis indicated that both extracts killed trophozoites by necrosis. In vivo studies showed that oral treatment with Pcc-WT-AE (4 and 8 mg/dose/hamster) completely prevented ALA development in 80% of animals, comparable to the effect of MTZ. In contrast, oral treatment with Pcc-KETc7-AE did not prevent lesions, but statistically reduced hepatomegaly, ALA size, and necrotic areas in tissue sections.

**Conclusion:**

The aqueous extracts from *C. papaya* embryogenic callus cultures analyzed here exhibit potent, yet variable, anti-*E. histolytica* activity in both in vitro and in vivo studies. The activity was nearly as effective as MTZ suggesting their potential use as a new, natural and safe oral treatment for amebiasis.

## Introduction

Parasitic diseases have always represented a public health problem in developing countries whose eradication is hampered by a variety of social, cultural, and economic factors. Moreover, the emergence of drug-resistant parasites adds another layer of complexity to this issue [1, 2]. Among these diseases, amoebiasis caused by the protozoan parasite *Entamoeba histolytica*, is one of the most frequent causes of diarrhea in children under 5 years old. The infection with this parasite can result in severe intestinal and extraintestinal illnesses including dysentery and amoebic liver abscess (ALA), the later responsible for most deaths from amoebiasis [3]. In Mexico, amoebiasis continues to pose a challenge, with notable incidence, whose persistence is exacerbated by the difficulties in implementing effective prevention and control measures [4–6]. The treatment of choice for amoebiasis is metronidazole (MTZ), a nitroimidazole that is very effective in combating the infection but also has some drawbacks such as numerous side effects (nausea, headaches, metallic taste and, in some cases, neurotoxicity and gastrointestinal disorders) [7] and the development of resistance by the parasite [8]. Resistance has been associated to nitroimidazole reductase activity encoded by the nim gene, which has been identified in cases of recurrent ALA [9].

Given the limitations of the currently available anti-amoebic treatments and the impact of ALA on human heath, the search for new drugs, particularly those derived from natural sources such as plants, has garnered substantial global interest [10, 11]. In a previous study, we reported the potent in vitro amoebicidal effect of extracts and alkaloids from the plant *Tabernaemontana arborea*; however, the compounds were unable to prevent the development of ALA in the golden hamster model, even when administered intraperitoneally [12]. Our interest focuses on the identification of natural compounds that, when administered orally or ingested, lead to extraintestinal protection against ALA without causing side effects. The antiparasitic properties of papaya (*Carica papaya*) has been attributed to various components, including papain, chymopapain, lysozyme, glycyl endopeptidase, cysteine proteinase, and benzyl isothiocyanate, which are concentrated in papaya latex and seeds [13–15]. Although methanolic extracts of *C. papaya* seeds have been shown to inhibit the in vitro growth of amoebae [16, 17], there is a lack of studies using aqueous extracts of the plant instead of solvent extracts as well as studies evaluating their effects in experimental animal models of amoeba infection. Currently, we have two papaya embryogenic callus cell lines, one obtained from non-genetically modified papaya (Papaya-Wild type) and another in which a 97 aa immunogenic peptide of the parasite *Taenia crassiceps* was expressed (Papaya-KETc7), which result in the loss of several papaya proteins and enrichment of others [13]. The different protein composition of these two callus cell lines could modify their anti-parasitic properties, making the comparison of their effects a strategy to target possible specific anti-parasite components of *C. papaya*. Indeed, although both callus cell lines decreased *T. crassiceps* viability in a similar extent, the Papaya-KETc7 cell line was more effective than Papaya-Wild type in decrease the in vitro viability of *Haemonchus contortus* L4 larvae [18]. Additionally, GK1, a peptide derived from KETc7, has been attributed adjuvant properties, positioning it as a promising molecule for exploring its protective capacity against infectious agents. Recently, we have established clonal papaya cell cultures obtained from the embryogenic calli of both cell lines (Pcc-WT, Pcc-KETc7), which maintained in controlled growing conditions, ensure a constant composition that provides a scalable and sustainable system to synthesize a product with pharmaceutical potential [19]. Herein, we prepared aqueous extracts of cell suspensions of Pcc-WT-AE and Pcc-KETc7-AE clones and evaluated their effect on the in vitro growth and viability of *E. histolytica* trophozoites as well as their effect by oral administration on the development of ALA in hamsters, comparing in both cases against the effect of MTZ.

## Materials and Methods

### Aqueous Extract from *C. papaya* Callus Cell Lines

Cell suspension from embryogenic callus of *C. papaya* untransformed (Papaya-Wild type) and transformed (Papaya-KETc7) were prepared following the procedure previously described [18]. Cultures from both lines were established from sub-cultured friable calluses in B5 medium as reported before [18]. The biomass, obtained as previously described [13], was pulverized using liquid nitrogen and dissolved in PBS at pH 7.4. The resulting suspensions were centrifuged at 15,500 x *g* for 30 min at 4 °C to remove insoluble debris. The supernatants (Pcc-WT-AE and Pcc-KETc7-AE), were then collected, and protein concentration was determined by measuring absorbance at 280 nm with a NanoDrop One spectrophotometer (Thermo Fisher Scientific). The authors declare that *C. papaya* calluses and cell suspensions were cultured in accordance with the institutional, national, and international guidelines, regulations and legislation.

### *E. histolytica* Culture

Axenic *E. histolytica* HM1:IMSS trophozoites were maintained as previously described [20]. Briefly, trophozoites were cultured in TYI-S-33 medium supplemented with 15% adult bovine serum (MicroLab) and 3% Diamond vitamin solution (Sigma-Aldrich) under microaerophilic conditions at 37 °C for 72 h. After 72 h of growth (log-phase), the trophozoites were harvested by chilling on ice for 5 min, followed by centrifugation at 165 x *g* for 5 min at 4 °C.

### Amoeba Viability Assay

MTT (3-[4,5-dimethylthiazol-2-yl]-2,5 diphenyl tetrazolium bromide) viability assays were performed as previously described [20]. In brief, trophozoites were harvested from cultures after 72 h, as detailed above, and seeded into 96-well culture plates at a density of 2 × 10^4^ trophozoites in 200 µl of fresh TYI-S-33 medium. Immediately, 10 µl of PBS (control) or 10 µl of Pcc-WT-AE or Pcc-KETc7-AE at concentrations between 50 and 500 µg/ml were added to each well. Additionally, MTZ (Sigma-Aldrich) was also tasted at concentration between 0.01 and 100 µg/ml. The plates were then incubated at 37 °C for 24 h. Posteriorly, 80 µl of MTT (1 mg/ml in PBS; Sigma-Aldrich) was added to each well, and the plates were incubated in the dark at 37 °C for 1 h. After incubation, the plates were centrifuged at 165 x *g* for 5 min, and the supernatant was carefully removed, leaving 100 µl per well. Then, each well was added with 100 µl of a pre-warmed sodium dodecyl sulfate (15%, BioRad) with hydrochloric acid (0.01 N) solution, and the contents were homogenized to dissolve the formazan crystals. Finally, absorbance at 595 nm was measured within 15 min using a Multiskan FC plate reader (Thermo Scientific). Experiments were performed three times in triplicate (*n* = 9).

### Apoptosis/Necrosis Assessment

To determine whether the aqueous extracts induce apoptosis or necrosis in *E. histolytica* trophozoites, a previously established methodology from our group was employed with some modifications [21]. Briefly, trophozoites were cultured at 2 × 10^4^ cells per 200 µl of fresh TYI‑S‑33 medium in 96-well culture plates, in quadruplicate. Amoebae were treated with the concentration corresponding to the IC_50_ of each extract and incubated for 24 h at 37 °C. Treated trophozoites were chilled on ice for 10 min, collected in tubes, and washed with PBS. Amoebae cultured for 24 h at 37 °C without any treatment were used as the viability control. Amoebae cultured for 24 h at 37 °C without extract treatment but incubated at 56 °C for 30 min were used as necrosis control. Trophozoites treated with 1 mM H_2_O_2_ for 2 h were used as a positive control for apoptosis. Trophozoites (8 × 10^4^) were resuspended in 195 µl of 1x Annexin V binding buffer (Enzo), and 5 µl of Annexin V-FITC (Enzo) were added. After 10 min of incubation, cells were washed and resuspended in 400 µl of binding buffer supplemented with 500 nM Sytox Green^®^ (Thermo Fisher). Parasites were incubated for 10 min at room temperature in the dark, and 100 µl of the cell suspension were dispensed per well. Fluorescence was measured from the bottom of the well using a Synergy HTX microplate reader (BioTek) with excitation/emission filters set at 485/528 nm. Experiments were performed three times in triplicate (*n* = 9).

### Amoebic Liver Abscesses Induction and Treatment

**Fig. 1:**
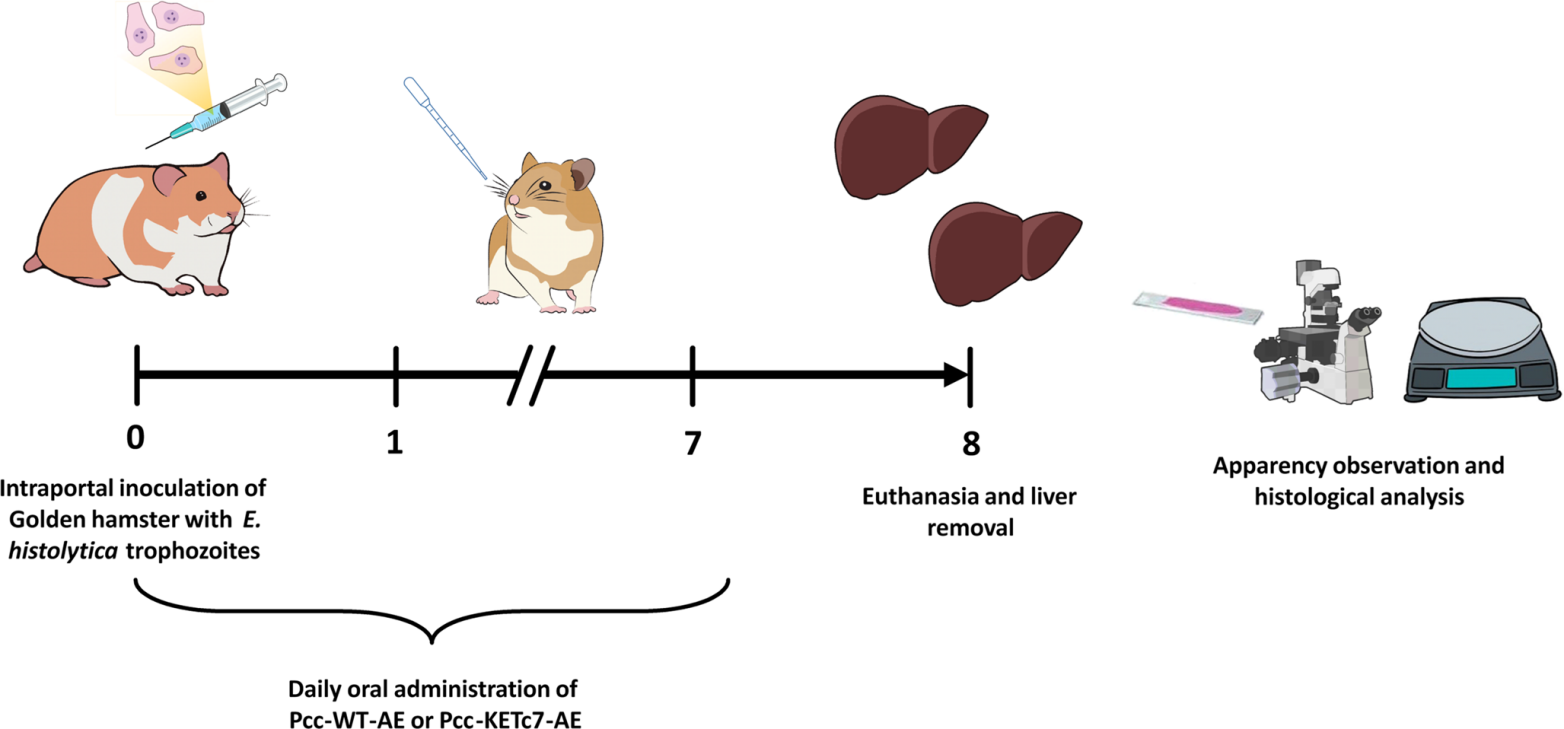
Induction of ALA and treatment protocol with soluble extracts from *C. papaya* calluses. Male hamsters (6 weeks-old) were inoculated via the intraportal route with 1 × 10^6^*E. histolytica* trophozoites. The animals were administered 400 µl of Pcc-WT-AE or Pcc-KETc7-AE containing either 4 mg or 8 mg of the extract starting at day 0 and repeated daily for 7 days. Sham animals received only 400 µl PBS. On the eighth day, the animals were euthanized and their livers were collected for weighing and histological examination

Male Syrian golden hamsters (*Mesocricetus auratus*) 6 weeks-old and approximately 100 g weight were maintained free of pathogens with water and food *ad libitum*. Infection was performed as we described previously [12], in brief, the animals were anesthetized with sodium pentobarbital (Pet’s Pharma), the peritoneal cavity opened by surgical laparotomy, and the portal vein exposed by removing the intestines from the abdominal cavity. A volume of 100 µl of parasites (1 × 10^6^ trophozoites) were directly inoculated into the portal vein bloodstream, the site of inoculation immediately occluded with gel foam pads, the intestines returned to the peritoneum and the abdomen sutured using vyclil 4 − 0. Before the animals regained consciousness (1–2 h post-inoculation), they received an oral administration of 400 µl PBS containing 4 or 8 mg total protein of either Pcc-WT-AE or Pcc-KETc7-AE (*n* = 5 per group) using an intragastric canula in previously Sevorane sedated animals. Control groups of infected hamsters (*n* = 5 per group) received MTZ 4 mg in 400 µl PBS as positive control of treatment or 400 µl PBS alone as sham control, all by oral route as above. Treatment was repeated daily for seven days, and on the eighth day, the hamsters were euthanized using excess of anaesthesia. The livers were excised, weighed, and abscesses pieces fixed in 4% paraformaldehyde for histology. Tissue sections of 5 μm were obtained in a microtome and Periodic Acid-Schiff (PAS) stained for microscopy (Fig. 1). The protocol was approved by the Institutional Animal Care and Use Committee of the Faculty of Medicine, UNAM with identification number CICUAL 5427.

### Necrotic Area and Parasite Burden Quantification

The necrotic area in liver tissue sections was quantified using the open-source software FIJI (ImageJ v1.53c). PAS-stained images were acquired under brightfield microscopy using a 10X objective and saved in TIFF format. For each sample, three non-overlapping fields were analyzed. A scale bar embedded in each image was used to calibrate the pixel-to-micrometer ratio in FIJI. Necrotic areas (defined as regions lacking cellular structure, exhibiting cellular debris and tissue disintegration) were manually delineated using the “Freehand Selections” tool. Each selected region was added to the ROI Manager, and its area was recorded using the “Measure” function. The measured area of necrosis was then expressed as a percentage relative to the total area of the image field. Results were reported as the percentage of necrotic area per image and averaged across the three analyzed fields for each sample.

The parasite burden in liver tissue sections was determined by manually counting *E. histolytica* trophozoites in images of PAS-stained tissue acquired under brightfield microscopy using a 40X objective. For each sample, four representative, non-overlapping fields were analyzed. PAS staining allowed clear identification of *E. histolytica* trophozoites, which exhibited an intense pink coloration characteristic of this stain. Using FIJI (ImageJ v1.53c), the total area of each image field was calculated by calibrating the pixel-to-micrometer ratio based on the embedded scale bar. Trophozoites were manually counted based on their staining pattern and morphology. The number of parasites was then normalized to the field area and expressed as amoebae per square millimeter (amoebae/mm²).

### Statistical Analysis

Graphs represent the mean ± standard deviation. Data were analyzed using GraphPad Prism 8.0.1 software, employing one-way ANOVA with Tukey’s post hoc test or the Kruskal–Wallis test followed by Dunn’s post hoc correction for multiple comparisons. The IC_50_, defined as the concentration of the compounds required to reduce trophozoite viability by 50% relative to the untreated control, were calculated from dose-response curves using non-linear regression analysis. Changes in infection percentage rate were determined using Fisher’s exact test. A *p* value ≤ 0.05 was considered statistically significant.

## Results

### *C. papaya* Aqueous Extracts Reduce *E. histolytica* Trophozoites Viability

**Fig. 2:**
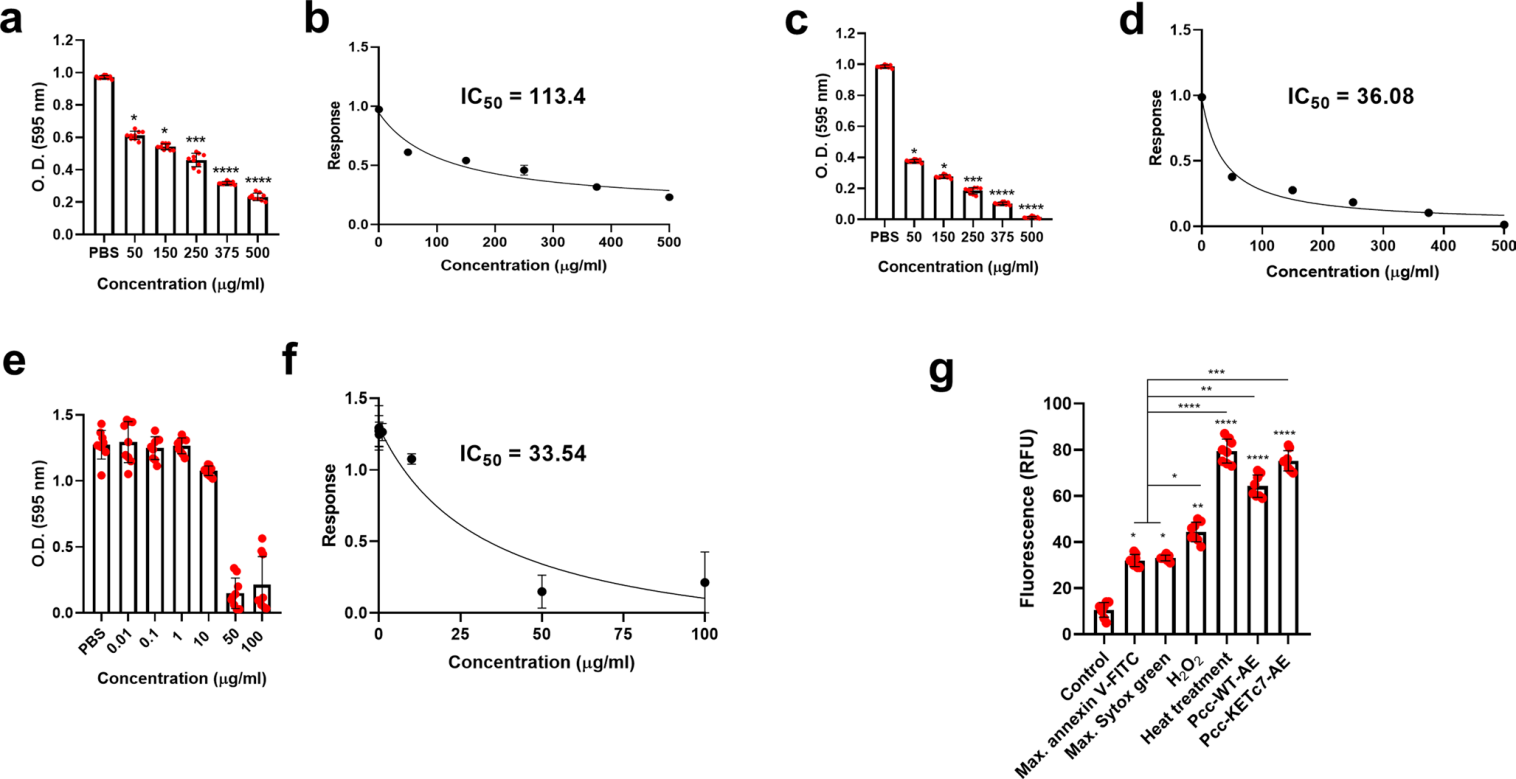
Effect of Pcc-WT-AE and Pcc-KETc7-AE on the viability of *E. histolytica* cultures. Trophozoites (2 × 10^4^)/well were cultured in the presence of (**a**) Pcc-WT-AE at crescent concentrations (50–500 µg/ml) for 24 h at 37 °C. After incubation, MTT solution was added to the wells for 1 h at 37 °C and subsequently, 60 °C pre-heated SDS–HCl was added to lyse the cells and dissolve formazan crystals. Absorbance was measured at 595 nm within 15 min and IC50 was determined (**b**). The response to increasing concentrations of Pcc-KETc7-AE was similarly evaluated (**c**), and its corresponding IC_50_ was determined (**d**). Additionally, the effect of metronidazole (MTZ) was tested under the same conditions (**e**), and its IC_50_ value was also calculated (**f**). To determine the type of cell death induced by the aqueous extracts trophozoites were treated for 24 h at 37 °C with the IC_50_ of Pcc-WT-AE or Pcc-KETc7-AE. Controls included untreated cells (viability), trophozoites exposed to 1 mM H₂O₂ for 2 h (apoptosis), and heat-treated cells at 56 °C for 30 min (necrosis). Heat-treated amoebae were also used to determine the maximum fluorescence values for Annexin V-FITC and Sytox Green^®^ individually (Max. annexin V-FITC and Max. Sytox green, respectively). After staining, fluorescence was measured using a Synergy HTX plate reader (485/528 nm). Values are presented as means ± SD of three independent experiments; RFU = Relative Fluorescence Units. *p < 0.05; **p < 0.01; ***p < 0.001; ****p < 0.0001

The viability of *E. histolytica* trophozoites cultures exposed to increasing concentrations of aqueous extracts Pcc-WT-AE and Pcc-KETc7-AE was tested. As shown in Fig. 2a, trophozoites treated with Pcc-WT-AE decreased their viability at all concentrations tested in a dose-dependent manner respect to the PBS treated culture (*p* < 0.05). Viability decreased more than 35% at the lowest concentration of 50 µg/ml and about 75% at the highest concentration of 500 µg/ml (Fig. 2a); IC_50_ of 113.4 µg/ml (Figs. 2b and 95% confidence interval: 73.32 to 185.0). Pcc-KETc7-AE also reduced the trophozoites viability in a dose-dependent manner (Fig. 2c). In comparison, Pcc-KETc7-AE at 50 µg/ml decreased amoebic viability in more than 50% and at 500 µg/ml killed all trophozoites; IC_50_ of 36.08 µg/ml (Figs. 2d and 95% confidence interval: 28.89 to 44.70). Additionally, the effect of MTZ at different concentrations on *E. histolytica* cultures was evaluated. A reduction in viability to nearly 10% compared to the untreated control was observed at a concentration of 50 µg/ml (Fig. 2e), and an IC_50_ of 33.54 µg/ml was calculated (Figs. 2f and 95% confidence interval: 21.73 to 54.02). These data demonstrate that Pcc-KETc7-AE exhibits amoebicidal activity in vitro that is comparable to that of MTZ.

### Necrosis was Induced in *E. histolytica* Trophozoites by Both Papaya Aqueous Extracts

We performed a fluorescence-based apoptosis/necrosis assay using Annexin V-FITC and Sytox green^®^. As expected, viable trophozoites (control) displayed low baseline fluorescence, indicating intact membranes and absence of phosphatidylserine exposure (Fig. 2g). Heat-treated trophozoites were incubated with either Annexin V-FITC or Sytox Green individually to establish the maximum fluorescence signal for each reagent. Hydrogen peroxide (H_2_O_2_) treatment showed a slight increase compared to the individual reagent controls, suggesting phosphatidylserine exposure on the outer leaflet of the membrane along with early membrane compromise, which is characteristic of apoptosis. In contrast, both Pcc-WT-AE and Pcc-KETC7-AE induced a marked increase in fluorescence intensity, reaching levels comparable to heat treatment (necrosis control). This suggests that both treatments trigger substantial loss of membrane integrity, consistent with necrosis.

### Oral Administration of *C. papaya* Aqueous Extracts Inhibits Hepatomegaly and the Development of ALA in Hamsters

**Fig. 3:**
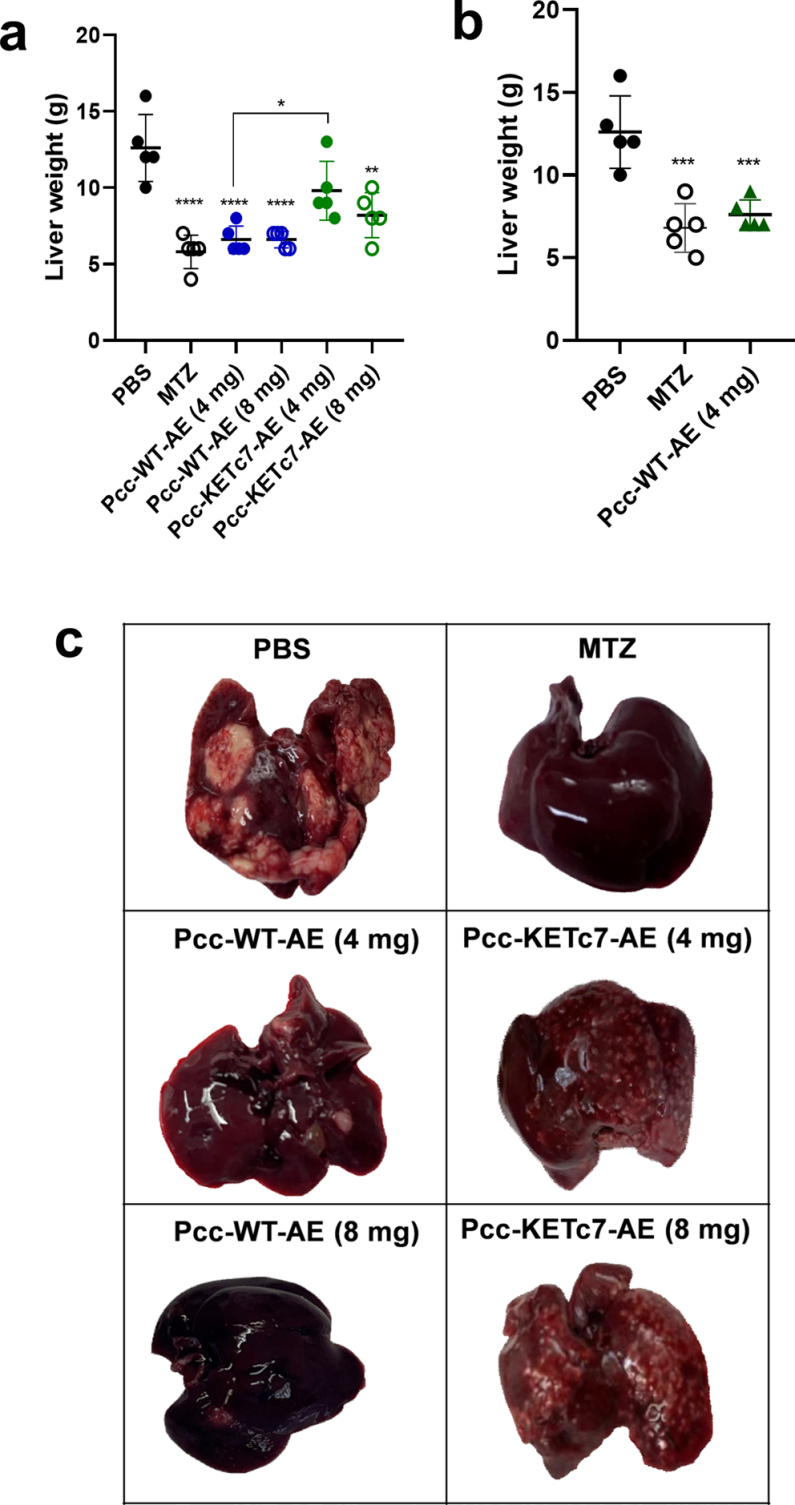
Effect of oral treatment with Pcc-WT-AE or Pcc-KETc7-AE on the development of ALA. *E. histolytica*-infected hamsters were euthanized, and their livers were extracted for analysis. (**a**) The liver weight was measured in sham animals (treated with PBS), the standard treatment MTZ (1 mg), Pcc-WT-AE or Pcc-KETc7-AE at doses of 4 or 8 mg. (**b**) The effect of Pcc-WT-AE at 4 mg dose was verified against sham and MTZ groups. (**c**) A representative image illustrating the appearance of livers from each of the groups. *p < 0.05; **p < 0.01; ***p < 0.001; ****p < 0.0001

Golden hamsters were intraportally inoculated with *E. histolytica* trophozoites and orally treated every day with Pcc-WT-AE or Pcc-KETc7-AE during 7 days, after which the animals were euthanized on day 8. As depicted in Fig. 3a, livers from infected animals receiving only PBS (sham control) showed a weight ranging from 10 to 16 g, which is two to three times the average weight of livers from infected animals treated with MTZ (about 6 g), which completely inhibited abscess development as expected (ALA inhibition positive control). Noteworthy, oral treatment of infected hamsters with Pcc-WT-AE at both tested doses (4 and 8 mg), as well as Pcc-KETc7-AE at the 8 mg dose, resulted in a statistically significant reduction in hepatomegaly, the livers of all animals showing a weight equal to or close to the average weight of the livers of hamsters treated with MTZ. Pcc-KETc7-AE at 4 mg dose also reduced hepatomegaly but at lesser extension (non-statistically significant for Pcc-KETc7-AE at 4 mg; *p* < 0.05 for Pcc-KETc7-AE at 8 mg; and *p* < 0.0001 for all other groups respect to PBS group). The potential of Pcc-WT-AE at 4 mg dose to completely prevent the development of hepatomegaly at the same level as MTZ was confirmed in a second experiment as shown in Fig. 3b.

Representative images showing the morphological characteristics of livers of each group studied are depicted in Fig. 3c. All 5 livers of sham hamsters displayed large and coalescence abscesses distributed across all hepatic lobes (Fig. 3c PBS and Table 1). On the other hand, as expected, none of the livers of the MTZ-treated hamsters developed ALA, maintaining the characteristic coloration and morphology of healthy, uninfected livers (Fig. 3c MTZ and Table 1). Interestingly, only 1 out 5 animals in each group treated orally with 4 mg or 8 mg of Pcc-WT-AE developed ALA, indicating a amoebicidal protection rate of 80% with no differences in efficiency between both doses tested (Fig. 3c; Pcc-WT-AE 8 mg). Furthermore, the only animal in each group that developed ALA showed few, small abscesses, with no evidence of coalescence (Fig. 3c; Pcc-WT-AE 4 mg). In contrast to what was observed with Pcc-WT-AE, all animals treated with 4 mg or 8 mg of Pcc-KETc7-AE developed ALA (no sterilizing protection was provided), but unlike sham animals (treated with PBS), the number and size of abscesses were reduced in all livers with little or no evidence of coalescence (partial protection in 100% of animals; Fig. 3c KETc7 4 and 8 mg).

**Table 1:** Infection/protection rate against ALA development in hamsters treated with Papaya aqueous extracts

Group	ALA development rate: ALA/total (%)	Sterilizing protection^a^		Partial protection^c^ (%)
		(%)	*p* value^b^ vs. PBS	
Sham (PBS)	5/5 (100)	0	NA	0
MTZ (1 mg)	0/5 (0)	100	0.0079	NA
Pcc-WT-AE (4 mg)	1/5 (20)	80	0.0476	20
Pcc-WT-AE (8 mg)	1/5 (20)	80	0.0476	20
Pcc-KETc7-AE (4 mg)	5/5 (100)	0	> 0.9999	100
Pcc-KETc7-AE (8 mg)	5/5 (100)	0	> 0.9999	100

^a^ Total inhibition of ALA development determined in tissue section of livers

^b^ Fisher’s exact test

^c^ Partial inhibition of ALA development denoted by reduction of hepatomegaly, ALA size and necrotic areas in tissue sections

### Oral Treatment with Pcc-WT-AE Protects Hamsters of Liver Tissue Destruction by Amoeba and Inflammatory Infiltrates

**Fig. 4:**
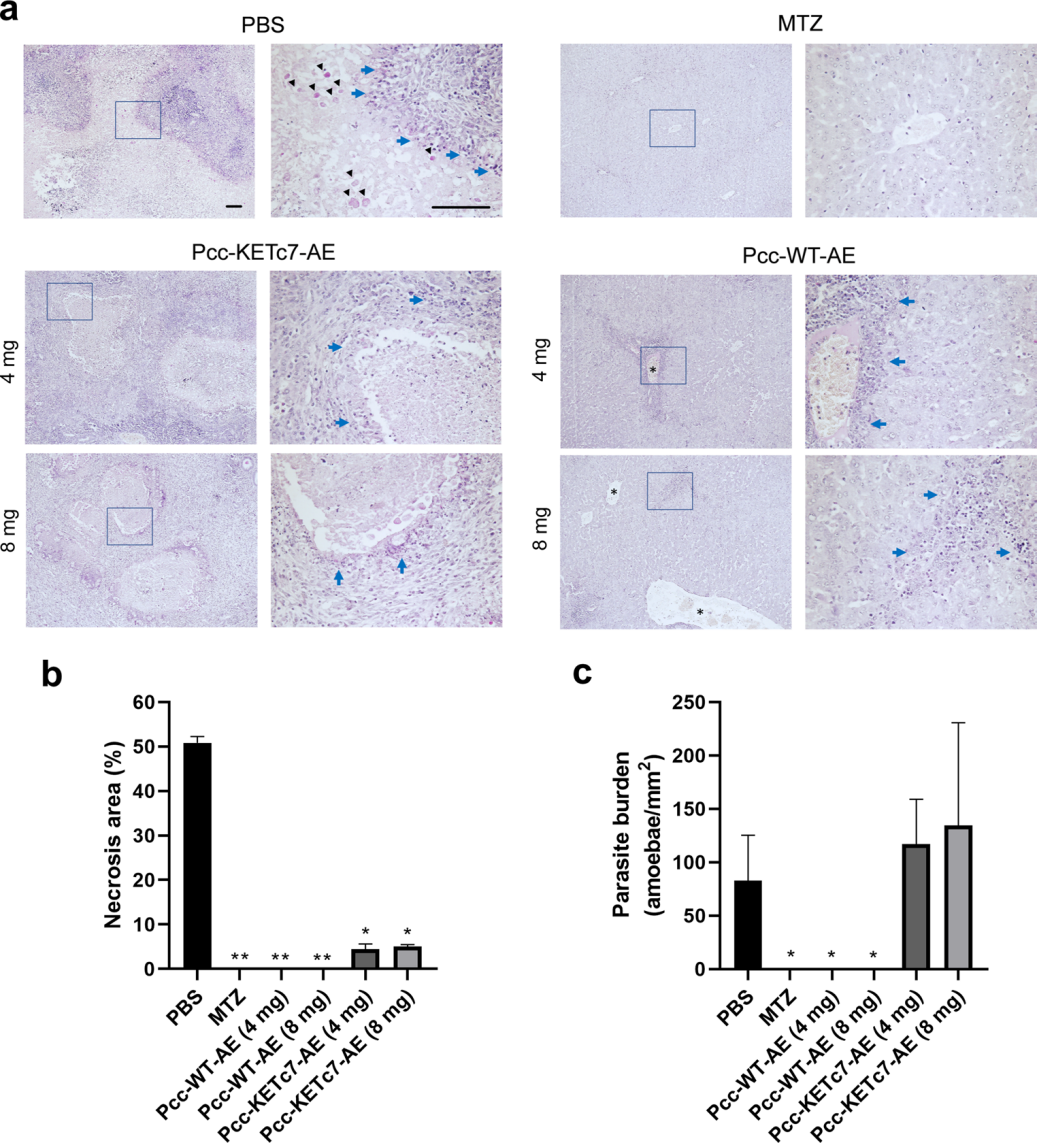
Effect of oral treatment with Pcc-WT-AE or Pcc-KETc7-AE on liver tissue destruction and inflammation during ALA development. (**a**) Liver samples from treated and sham hamsters were embedded in paraffin for subsequent PAS staining. Representative images from each group are shown at 10X and 40X magnifications (the magnified zone is indicated by a blue box.). Sham (PBS); metronidazole (MTZ); Pcc-WT-AE at 4 mg and 8 mg; and Pcc-KETc7-AE at 4 mg and 8 mg; Trophozoites (black arrowhead), inflammatory infiltrate (blue arrow) and sinusoidal congestion (asterisk) are indicated. Scale bars represent 100 μm. (**b**) Necrotic areas were manually outlined in PAS-stained images (10X) using FIJI, based on loss of tissue structure and cellular debris, and expressed as a percentage of the total image area. (**c**) Parasite burden was assessed by manually counting *E. histolytica* trophozoites (40X) identified by their intense pink PAS staining. Counts were normalized to the image area and reported as amoebae/mm². *p < 0.05; **p < 0.01

Histological sections of hamster livers were stained with PAS for detailed microscopic examination. Fig. 4adepicts the histology of a liver section from an infected sham animal (PBS). Typical areas of extensive necrosis (defined as disorganized tissue, with pale or absent PAS reactivity and indistinct cellular boundaries, accumulation of cellular debris, and loss of nuclear detail) surrounded by abundant inflammatory infiltrate are observed. At higher magnification, *E. histolytica* trophozoites in the necrotic area are clearly observed (black arrowheads), surrounded by abundant polymorphonuclears leucocytes (PMN; blue arrows). In animals infected and treated with MTZ, the hepatic parenchyma and cords show normal morphology, without evident damage or the presence of trophozoites with inflammatory cells. Animals treated with 4 or 8 mg Pcc-KETc7-AE showed some necrotic areas with mild inflammatory infiltrate but, in contrast to sham animals, the necrosis did not extend throughout the liver tissue, but was localized. On the other hand, the livers of animals treated with 4 mg or 8 mg Pcc-WT-AE showed nor necrotic areas neither amoebae, but discrete and infrequent areas of inflammatory infiltrate (blue arrow) and mild degree of sinusoidal congestion (asterisk), manifested as an increase in the diameter of some blood vessels, were observed.

Figure 4b and c show the percentage of necrosis areas and parasite burden calculated in liver tissue sections from hamsters. Animals infected and treated with PBS (sham) showed an extension of necrosis close to 50% and a count of amoebic trophozoites close to 100/mm^2^, whereas no detectable necrosis or amoebic trophozoites were observed in the Pcc-WT-AE groups orally treated with 4 or 8 mg/doses and the MTZ-treated group (*p* < 0.01 and *p* < 0.05 vs. PBS group, respectively), suggesting sterilizing protection by Pcc-WT-AE similar to MTZ. On the other hand, tissue sections from hamsters orally treated with Pcc-KETc7-AE at both doses, although showing about 10 times less necrotic area than sham animals (*p* < 0.05), showed the same count of amoebic trophozoites in the tissue as those, suggesting only partial protection from the development of ALA.

## Discussion

*E. histolytica* remains as one of the parasites with the highest incidence worldwide showing prevalence of up to 72% in certain regions [22, 23]. Since the parasite is transmitted by the fecal-oral route, inadequate food preparation practices and limited access to clean water sources are relevant factors for the high frequency of cases, particularly in developing countries where these conditions prevail [24, 25]. Amoebiasis can present with symptoms of varying severity ranging from diarrhea to serious complications such as amoebic colitis, amoebic dysentery, or amoebic liver abscesses [3]. The treatment of choice is MTZ, a low-cost drug that resolves the disease in most cases; however, its side effects in patients and the development of resistance by the parasite remain important drawbacks [8, 26–28]. Therefore, the search for alternative treatments to combat amoebiasis remains a topic of pivotal interest.

In this study, we investigated the amoebicidal potential of aqueous extracts obtained from cell cultures of two embryogenic callus lines of *C. papaya* previously reported by our group [29], the non-transformed line Papaya-WT and the genetically transformed line expressing the KETc7 peptide, Papaya-KETc7. Our group has previously reported differences in the ability of the lines to confer protection to *H. contortus*, which may be attributable to differences in protein expression and enrichment, which we have previously reported [18]. In this work, our findings indicate that the aqueous fraction of both callus lines exhibited dose-dependent amoebicidal activity in vitro, with Pcc-KETc7-AE being more effective against the parasite than Pcc-WT-AE (IC_50_ 36 vs. 113.4 mg/ml, respectively, at 24 h post-exposure). These results demonstrate that a simple aqueous preparation of *C. papaya* callus is capable of killing *E. histolytica* trophozoites in vitro, a result that is comparable to those previously reported with methanolic extracts of seeds [16, 17]. In fact, the IC_50_ obtained for Pcc-KETc7-AE exceeds that previously reported for one of those methanolic extracts (153 µg/ml; Calzada et al., 2006), being rather closer to the effect we observed with Pcc-WT-AE (113.4 µg/ml). In the other report, the methanolic extracts killed all amoebae at a concentration of 125 µg/ml [16], which is four times lower than the concentration of Pcc-KETc7-AE required to achieve the same effect. However, the use of different *E. histolytica* strains (DS-4 868 vs. HM1:IMSS used in this study), different methods to assess amoebic viability (microscopic counting vs. MTT assay used here, which is much more reliable) and the use of different plant parts (seed vs. callus), which could contain different concentrations of papain, make comparison in this case challenging. It is unknown why Pcc-KETc7-AE was more efficient than Pcc-WT-AE against the amoeba in vitro, but it could be attributed to the protein differences between them. As previously reported by our group, the transgenic clone expresses much less proteins than the WT in 2D proteomic analysis (50 vs. 109, respectively) [18]. However, Pcc-KETc7-AE showed two spots, absent in Pcc-WT-AE, whose MW and PI correspond to chymopapain, the main cysteine ​​proteinase in papaya [18]. This observation raises the possibility that chymopapain may be responsible for the higher amoebicidal effect observed with Pcc-KETc7-AE. Further experiments are needed to evaluate this proposal. The aqueous extracts of *C. papaya* callus cell cultures used in this work represent an advantage over previously used extracts. On the one hand, these extracts are very promising as an alternative that outperforms solvent extracts by obvious reasons, and on the other, the availability of cultured papaya cells represents an advantage of yielding consistent results by eliminating the variability expected between individual plants, even within the same species [19, 30] as well as it mitigates the differences that can arise due to seasonal changes or the age of the specimens [31].

The observations on the amoebicidal effect of aqueous extracts in vitro, unlike others reports, were in this case complemented by studies on their effect in an experimental model, particularly on the development of ALA, the most severe and lethal manifestation of extraintestinal amoebiasis in humans [3]. Interestingly, daily oral treatment with any of the aqueous extracts of papaya (at 4 or 8 mg/dose) affected the development of ALA in infected hamsters, either by completely inhibiting its development as in the case of Pcc-WT-AE, that is, conferring sterilizing protection, or by decreasing the degree of ALA development (hepatomegaly and size) as in the case of Pcc-KETc7-AE. It is noteworthy that Pcc-WT-AE showed close protective potential against ALA as MTZ (80 vs. 100%, respectively), the drug of choice for the treatment of intestinal and extraintestinal amoebiasis as it is the most efficient amoebicide known. The protection in an extraintestinal compartment (liver) conferred by the oral administration of the aqueous extracts suggests that papaya components are absorbed in the intestine and pass to the liver, proposal supported by an in silico study showing that *C. papaya* antimicrobial-active compounds can be pharmacodynamically and pharmacokinetically stable once absorbed [32]. Once in the liver, the *C. papaya* compounds could directly kill amoebic trophozoites as suggest our in vitro studies reported here. However, the probability that papaya components exert an immunomodulatory effect that regulates inflammation in the liver and thus prevents the development of ALA cannot be ruled out, especially if we consider that no inflammatory infiltrates were observed in the livers from animals sterilely protected with Pcc-WT-AE and that an exacerbated immune response may be decisive in the development of ALA in this golden hamster model [33]. The reason why the extract of the WT clone performed better in vivo while the extract of the transgenic one was slightly superior in vitro could also have to do with the differences in protein expression between the extracts, mentioned above. In this case, the higher number of proteins in Pcc-WT-AE could have contributed to protection during infection due to the presence of proteins that better resisted the onslaught of gastric juices as they passed through the intestine into the bloodstream. Another possibility is that some of these proteins were processed into products more active against the amoeba. Studies to identify the components of Pcc-WT-AE that confer sterilizing protection against ALA are being carried out in our laboratory.

Our results of protection against ALA by administering the extracts orally are encouraging as they suggest that the treatment could work even better to control intestinal amoebiasis, experiments that will be further carried out in our laboratory. In this regard, there are two previous studies of protection against intestinal amoebiasis using extracts of *C. papaya* that were carried out in immunosuppressed mouse models (CD1 and Swiss albino) orally infected with trophozoites and cysts isolated from feces of patients. Although both studies reported a protective effect of fruit and seed extracts against the establishment of the parasite in the intestine of mice, they have a series of limitations that obscure the results. These include the use of “isolated” parasites that were not characterized at the time (no differentiation was made between *E. histolytica* and *Entamoeba dispar*, the latter being non-pathogenic), lack of certainty about the reproducibility of the model in terms of the degree of intestinal colonization and mucosal invasion, and the use of immunosuppressed animals, which together limit the robustness of the results [34–36]. In comparison, our study provides a higher level of certainty by using axenic trophozoites of the widely studied *E. histolytica* strain HM1:IMSS, as well as the experimental amoebiasis model most used for protection studies. Although no cytotoxicity assays were performed in the present study, the use of aqueous extracts from *C. papaya* (a plant widely regarded as safe) suggests a low likelihood of toxicity. Nevertheless, the lack of formal toxicity assessment represents one of the main limitations of this work and will be addressed in future studies. Our results contribute to the documented efficacy of *C. papaya* extracts against other protozoa in vivo, such as the ability of seeds chloroform extract (50 to 75 mg/kg) of reducing bloodstream *Trypanosoma cruzi* load in BALB/c mice [14] or the capacity of leaves aqueous extract (350 mg/kg) of reducing parasitemia and restoring hepatic cell damage in adult mice inoculated with *Plasmodium berghei* [37], to cite some examples.

## Conclusion

Our results demonstrate the amoebicidal properties of *C. papaya* callus extracts, specifically Pcc-WT-AE and the genetically modified Pcc-KETc7-AE. Both extracts exhibited potent in vitro amoebicidal activity, highlighting the potential of *C. papaya* as a therapeutic agent against *E. histolytica*. However, while Pcc-WT-AE showed promising results in reducing ALA formation in vivo, Pcc-KETc7-AE did not provide effective protection, possibly influenced by genetic modifications and differences in bioavailability. These findings point to Pcc-WT-AE merging as a new natural strong candidate for developing an orally administered antiparasitic agent effective against extraintestinal amoebiasis at a comparable level as MTZ without their adverse effects.

## Data Availability

No datasets were generated or analysed during the current study.
